# Depression, Anxiety, Stress, and Their Associations With Quality of Life in a Nationwide Sample of Psychiatrists in China During the COVID-19 Pandemic

**DOI:** 10.3389/fpsyg.2022.881408

**Published:** 2022-06-23

**Authors:** Mengdie Li, Lei Xia, Yating Yang, Ling Zhang, Shujing Zhang, Tingfang Liu, Yuanli Liu, Nadine J. Kaslow, Feng Jiang, Yi-lang Tang, Huanzhong Liu

**Affiliations:** ^1^Department of Psychiatry, Chaohu Hospital of Anhui Medical University, Hefei, China; ^2^Department of Psychiatry, School of Mental Health and Psychological Sciences, Anhui Medical University, Hefei, China; ^3^Department of Psychiatry and Behavioral Sciences, Emory University, Atlanta, GA, United States; ^4^School of Health Policy and Management, Chinese Academy of Medical Sciences and Peking Union Medical College, Beijing, China; ^5^Public Health School, Chinese Academy of Medical Sciences and Peking Union Medical College, Beijing, China; ^6^Healthcare Management and Evaluation Research Center, Institute of Health Yangtze River Delta, Shanghai Jiao Tong University, Shanghai, China; ^7^School of International and Public Affairs, Shanghai Jiao Tong University, Shanghai, China; ^8^Atlanta Veterans Affairs Medical Center, Decatur, GA, United States

**Keywords:** psychiatrists, depression, anxiety, stress, quality of life, COVID-19

## Abstract

**Objectives:**

The COVID-19 pandemic has negatively affected wellbeing. However, the impact on the mental health status of Chinese psychiatrists and their relationship with quality of life (QOL) has not been examined.

**Methods:**

This was a national cross-sectional survey performed between January 11 and March 15, 2021. Demographic and work-related data were collected anonymously using an online questionnaire. Common mental health symptoms and QOL were assessed using the Depression Anxiety Stress Scale-Chinese version and the World Health Organization Quality of Life Schedule-Brief, respectively.

**Results:**

A total of 3,783 psychiatrists completed this questionnaire. The prevalence of depressive, anxious, and stress symptoms were 26.7% (95%CI = 25.3–28.1%), 24.1% (95%CI = 22.8–25.5%), and 11.6% (95%CI = 10–12%), respectively. Moreover, 11.1% of the participants endorsed both depressive and anxious symptoms, and 8% endorsed symptoms in all three domains. Binary logistic regression showed that aged 30–39 years (OR = 1.5, *P* = 0.03), male gender (OR = 1.2, *P* = 0.04), single (OR = 1.44, *P* = 0.01), and having a negative perception of the COVID-19 on healthcare (OR = 2.34, *P* <0.001) were factors associated with higher levels of depressive symptoms. Participants who were divorced and widowed (OR = 1.56, *P* = 0.03), worked more than 4 night shifts/month (OR = 1.33, *P*<0.001) and/or longer working years (OR = 1.71, *P* < 0.001), and had a negative perceived impact of the COVID-19 on healthcare (OR = 2.05, *P* < 0.001) were more inclined to experience anxious symptoms. In addition, psychiatrists with high QOL scores had lower odds of experiencing depressive, anxious, and stress symptoms (all *P* < 0.001). Multivariate analysis showed that the presence of each of the three types of mental health symptoms was independently associated with lower QOL (all *P* < 0.05). In addition, the depression × anxious × stress interaction was significantly correlated with lower QOL (*P* < 0.05).

**Conclusion:**

Approximately one-fourth of psychiatrists in China experienced depressive and anxious symptoms during the COVID-19 pandemic, and more than one-tenth reported stress. Mental health symptoms were significant contributors to lower QOL. The psychological wellbeing of psychiatrists during the pandemic requires more attention, and interventions are needed to improve the psychological wellbeing and QOL of physicians who care for individuals with mental disorders.

## Introduction

The coronavirus disease (COVID-19) has had an unprecedented and devastating impact on almost all aspects of the society, including healthcare (Pang et al., 2020; Luo et al., 2020), economy (Hertz-Palmor et al., 2020), education (Kaufman et al., 2021), environment (El Kenawy et al., 2021), and others. Compared with other occupations, healthcare workers have faced greater challenges during the pandemic, including increased risk of infection and workload, as well as heightened exposure to death, trauma, or violence. These experiences in turn impact healthcare workers' physical and psychological wellbeing (Johnson et al., 2021).

Reports from multiple countries involving professionals from different backgrounds have shown that, during the pandemic, healthcare professionals have experienced a range of mental health problems, most notably depression, anxiety, stress, or posttraumatic stress disorder (Lai et al., 2020; Azoulay et al., 2021; Hirten et al., 2021). A cross-sectional study in China revealed that physicians were at a higher risk of severe depression than were nurses and frontline healthcare workers who had more severe insomnia than the second-line healthcare workers (Lai et al., 2020). Unfortunately, this study did not include mental health professionals, such as psychiatrists, as they historically have not been on the frontlines when dealing with infectious diseases. Psychiatrists, however, have played a major role in supporting frontline healthcare professionals during the COVID-19 crisis through the provision of support groups and individual mental health services (Viswanathan et al., 2020; Cormi et al., 2021). The limited relevant data indicate that in the acute phase of the pandemic, levels of mental health symptoms in mental health professionals including psychiatrists tended to be somewhat lower than in the general public in Canada (Brillon et al., 2021) and somewhat lower than physicians from other specialties in Croatia (Jokic-Begic et al., 2020). Moreover, there is some evidence that compared with non-psychiatric physicians, psychiatrists tended to rely more heavily on substance misuse to cope with their pandemic-related stress (Jokic-Begic et al., 2020).

Work-related psychological stress and mental health symptoms often lead to poor quality of life (QOL) (Ruo et al., 2003; Wilmer et al., 2021), as reflected in people's physical, psychological, and social functioning. A study conducted in Saudi Arabia found that healthcare staff in intensive care units (ICU) and emergency units during the pandemic reported low QOL (Maqsood et al., 2021). However, scarce data are available on the effects of the COVID-19 pandemic on the QOL in psychiatrists working in public hospitals. Furthermore, the extent of psychological symptoms and their correlations with QOL in this professional group have not been examined.

Therefore, this study, conducted in a unique time and based on a nationally representative sample, was set to survey the rates of common mental health symptoms, such as depressive, anxious, and stress symptoms, and the QOL among psychiatrists in China during the pandemic. We also explored the comorbidity of different symptoms and the factors associated with those symptoms and QOL. Finally, we examined the relations between mental health symptoms and QOL.

## Materials and Methods

### Study Design and Participants

The study was part of the 2021 National Hospital Performance Evaluation Survey (NHPES) between January 11 and March 15, 2021, sourced by the National Health Commission of China (NHCC). We selected all provincial tertiary psychiatric hospitals in 31 provinces in mainland China. Two provinces (Tibet and Gansu) were not included because there were no tertiary psychiatric hospitals at the time of the survey. The study was reviewed and approved by the Ethics Committee of Chaohu Hospital of Anhui Medical University (202002-kyxm-02). The study was conducted *via* WeChat, a popular social media app in China.

It is worth noting that while the COVID-19 first started in China in December 2019, at the time of this survey (January to March 2021), which was rapidly spreading in most other parts of the world, the pandemic had been mostly under control in mainland China. Therefore, no specific mitigation strategies related to COVID-19 were needed. Furthermore, the survey was mostly conducted online, and minimal in-person contact was needed.

### Questionnaires

The questionnaire had three parts. The first part included demographic information and working-related information such as age, sex, marital status, education level, monthly income, number of night shifts per month, and working years. In addition, COVID-19-related issues were also assessed: (1) whether or not they had the frontline work experience with COVID-19 patients (yes/no); (2) perceived impact of the COVID-19 on medical work (negative impact: the intention to leave and change careers; positive impact: prefer to be a medical worker and love the career more; minimal or neutral impact) (Byrne et al., 2021).

The second part was the Depression Anxiety Stress Scale-Chinese version (DASS-C21), which was used to assess depressive, anxious, and stress symptoms in the past week (Lovibond and Lovibond, 1995). The 21-item self-reported scale consists of three subscales (seven items for each), namely, DASS-depression, DASS-anxiety, and DASS-stress. Participants responded to each question by using a 4-point (0–3) Likert-type scale (ranging from 0 = “did not apply to me at all” to 3 = “applied to me very much or most of the time”). The score was calculated by adding up the individual items on each subscale and multiplying them by two, with the subscale scores ranging from 0 to 42 and total scores from 0 to 126 (Yohannes et al., 2019). The DASS-C21 had good internal consistency among the items in the present sample (Cronbach's α = 0.951). In addition, DASS-depression (Cronbach's α = 0.906), DASS-anxiety (Cronbach's α = 0.854), and DASS-stress (Cronbach's α = 0.888) subscales also showed a strong reliability coefficient as Cronbach's α ≥ 0.7 is usually considered “acceptable” (Schober et al., 2021). The higher the score, the more severe the depressive, anxious, or stress symptoms in each subscale (Eyice Karabacak et al., 2021). DASS-depression scores > 9, DASS-anxiety scores > 7, and DASS-stress scores > 14 are considered as “having depression, anxiety, or stress symptoms” (Fawzy and Hamed, 2017).

The third part was the Chinese version of the World Health Organization quality of life Schedule-Brief (WHOQOL-BREF-CHN), an abbreviated version of the WHOQOL-100 that was developed by the WHOQOL Group in 1998 (The WHOQOL Group., 1998). It covers four domains (physical health, psychological, social relationships, and environment) and two separate items (general quality of life and general health state). In this study, overall QOL was assessed using the two separate items of the WHOQOL-BREF-CHN: (1) general quality of life, “How would you rate your quality of life?” (ranging from 1 = “very poor” to 5 = “very good”); (2) general health state, “How satisfied are you with your health?” (ranging from 1 = “very dissatisfied” to 5 = “very satisfied”), in which a higher score represents a better QOL (Li et al., 2021b). Although only two separate items were selected in our study, previous studies (Haag et al., 2017; Li et al., 2021b) found that the general quality of life and general health state were positively correlated with the scores of the four domains of WHOQOL-BREF-CHN, indicating that the two separate items of the scale had a good consistency with the four domains of the scale. Therefore, we chose these two separate items to evaluate the QOL of the overall sample. Furthermore, the WHOQOL-BREF-CHN is a valid and reliable tool to assess QOL (Cronbach's α = 0.78) (Kruithof et al., 2018).

### Statistical Analysis

Statistical analyses were executed in IBM SPSS 22.0 version for Windows. The Student's *t*-test was used and illustrated as mean ± standard deviation if normality distribution; if not, the Mann-Whitney *U*-test was utilized and expressed as median, quartile spacing (IQR). In our study, the Student's *t*-test was used for the univariate analysis of continuous variables, such as monthly income, general QOL, and general health status. Categorical variables were analyzed by chi-square tests and described as frequency and percentage. Normality distribution was detected by the one-sample Kolmogorov-Smirnov test for continuous variables. Binary logistic regression models (univariable and multivariable) were used to investigate the independent risk factors associated with depressive, anxious, and stress symptoms. In a multivariable logistic regression model, mental health problems (such as depressive, anxious, and stress symptoms) were the dependent variables, and the covariates were the variables that showed significant differences (*P* < 0.2) between the depression/no-depression, anxiety/no-anxiety, and stress/no-stress groups in the univariable analysis (Amu et al., 2021). The independent associations of depressive, anxious, and stress symptoms and their interaction with QOL were analyzed using multivariate analysis of covariance (MANOVA) after controlling for variables with striking group differences in univariable analyses. The QOL scores, such as general QOL scores and general health status scores, were used as the dependent variables (continuous variables), and depressive, anxious and stress symptoms were used as the independent variables (categorical variables). A proportionate Venn diagram quantified the prevalence of probable depressive, anxious, and stress symptoms among participants by https://hiplot.com.cn. For all tests (except univariable analysis), *P* < 0.05 was designated as the level of significance (2-tailed).

## Results

### Basic Features and the Prevalence of Probable Depressive, Anxious, and Stress Symptoms

All psychiatrists working at the selected 41 tertiary psychiatric hospitals (*N* = 4,899) were invited to participate in the study by submitting the online survey on WeChat, a multipurpose messaging App in China, either using their mobile phones or computers. The sample size of this study was calculated by PASS11. A sample size of 3,608 produces a two-sided 95% confidence interval (CI) with a distance from the mean to the limits that is equal to 0.140 when the estimated standard deviation is 4.290 (Zhang et al., 2021). A total of 3,973 responded to the survey (response rate = 81%), and 3,783 (77%) completed and were eligible for the statistical analysis. The demographic features of all psychiatrists are shown in Table 1.

**Table 1:** Basic features, occurrences of depressive, anxious and stress symptoms in 3,783 Psychiatrists in China.

**Variable**	**All sample** **(*N* = 3,783)**	**Depression** **(*N* = 1,011)**	**No depression** **(*N* = 2,772)**	**Univariable analysis**		**Anxiety** **(*N* = 913)**	**No anxiety** **(*N* = 2,870)**	**Univariable analysis**		**Stress (*N* = 416)**	**No stress (*N* = 3,367)**	**Univariable analysis**	
				***T/χ^2^***	***P***			***T/χ^2^***	***P***			***T/χ^2^***	***P***
**Age (years)** ^a^				11.94	0.008^**^			11.99	0.007^**^			15.18	0.002^**^
≤ 29	495 (13.1)	120 (24.24)	375 (75.76)			104 (21.01)	391 (78.99)			49 (9.90)	446 (90.10)		
30–39	1,770 (46.8)	506 (28.59)	1,264 (71.41)			467 (26.38)	1,303 (73.62)			229 (12.94)	1,541 (87.06)		
40–49	962 (25.4)	264 (27.44)	698 (72.56)			229 (23.80)	733 (76.20)			96 (9.98)	866 (90.02)		
≥50	556 (14.7)	121 (21.76)	435 (78.24)			113 (20.32)	443 (79.68)			42 (7.55)	514 (92.45)		
**Sex** ^a^				10.63	**<0.001** ^***^			2.63	0.105^*^			3.15	0.08^*^
Male	1,521 (40.2)	450 (29.59)	1,071 (70.41)			388 (25.51)	1,133 (74.49)			184 (12.10)	1,337 (87.90)		
Female	2,262 (59.8)	561 (24.80)	1,701 (75.20)			525 (23.21)	1,737 (76.79)			232 (10.26)	2,030 (89.74)		
**Marital status** ^a^				12.21	0.016^**^			7.50	0.112^*^			12.31	0.002^**^
Single	631 (16.7)	192 (30.43)	439 (69.57)			158 (25.04)	473 (74.96)			89 (14.10)	542 (85.90)		
Married	3,008 (79.5)	772 (25.66)	2,236 (74.34)			708 (23.54)	2,300 (76.46)			304 (10.11)	2,704 (89.89)		
Divorced and widowed	144 (3.8)	47 (1.56)	97 (3.22)			47 (32.64)	97 (67.36)			23 (15.97)	121 (84.03)		
**Education level** ^a^				41.31	<0.001^***^			35.51	<0.001^***^			7.23	0.065^*^
College degree/medical degree only	2,438 (64.4)	732 (30.02)	1,706 (69.98)			661 (27.11)	1,777 (72.89)			291 (11.94)	2,147 (88.06)		
Add on master degree	1,115 (29.5)	238 (21.35)	877 (78.65)			216 (19.37)	899 (80.63)			106 (9.51)	1,009 (90.49)		
Add on doctorate degree	230 (6.1)	41 (17.83)	189 (82.17)			36 (15.65)	194 (84.35)			19 (8.26)	211 (91.74)		
**^#^Monthly income (RMBs)^b^**	12.2 ± 7.6	10.54 ± 6.11	12.86 ± 7.96	8.41	<0.001^***^	10.16 ± 5.64	12.91 ± 7.99	9.64	<0.001^***^	9.83 ± 5.68	12.54 ± 7.73	6.91	<0.001^***^
**Number of night shifts per month** ^a^				32.37	<0.001^***^			52.40	<0.001^***^			32.29	<0.001^***^
≤ 4 times	2,840 (75.1)	692 (24.37)	2,148 (75.63)			603 (21.23)	2,237 (78.77)			603 (21.23)	2,237 (78.77)		
>4 times	943 (24.9)	319 (33.83)	624 (66.17)			310 (32.87)	633 (67.13)			310 (32.87)	633 (67.13)		
**Working years** ^a^				2.10	0.35			7.08	0.029^**^			3.54	0.171^*^
≤ 5 years	834 (22)	207 (24.82)	627 (75.18)			174 (20.86)	660 (79.14)			97 (11.63)	737 (88.37)		
6–10 years	828 (21.9)	222 (26.81)	606 (73.19)			217 (26.21)	611 (73.79)			103 (12.44)	725 (87.56)		
≥11 years	2,121 (56.1)	582 (27.44)	1,539 (72.56)			522 (24.61)	1,599 (75.39)			216 (10.18)	1,905 (89.82)		
**Frontline experience with COVID-19 patients** ^a^				0.24	0.62			0.25	0.62			0.03	0.87
Yes	887 (23.4)	243 (27.40)	644 (72.60)			223 (25.14)	664 (74.86)			101 (11.39)	786 (88.61)		
No	2,764 (73.1)	734 (26.56)	2,030 (73.44)			672 (24.31)	2,092 (75.69)			309 (11.18)	2,455 (88.82)		
**Perceived impact of the COVID-19**^a^ **on medical work**				248.94	< .001^***^			161.28	< .001^***^			164.81	< .001^***^
Negative	486 (12.8)	266 (54.73)	220 (45.27)			229 (47.12)	257 (52.88)			137 (28.19)	349 (71.81)		
Positive	1,443 (38.1)	262 (18.16)	1,181 (81.84)			273 (18.92)	1,170 (81.08)			109 (7.55)	1,334 (92.45)		
Minimal impact	1,722 (45.5)	449 (26.07)	1273 (73.93)			393 (22.82)	1,329 (77.18)			164 (9.52)	1,335 (77.53)		
**QOL score** ^b^													
General QOL	3.11 ± 0.78	2.63 ± 0.74	3.29 ± 0.73	24.54	<0.001^***^	2.67 ± 0.74	3.25 ± 0.74	20.52	<0.001^***^	2.51 ± 0.82	3.18 ± 0.75	17.27	<0.001^***^
General health status	2.82 ± 0.93	2.32 ± 0.83	3.01 ± 0.89	21.48	<0.001^***^	2.29 ± 0.83	3.00 ± 0.90	20.83	<0.001^***^	2.07 ± 0.79	2.91 ± 0.90	18.27	<0.001^***^

*Depression with a cutoff value 9, anxiety with a cutoff value 7, stress with a cutoff value 14*.

a *Chi-square test*.

b *t-test*.

# *Monthly income in thousands (RMBs), one US dollar = 6.3969 RMBs at the time of study*.

*Bold values*:

* *P < 0.2*,

** *P < 0.05*,

*** *P < 0.001*.

**Figure 1:**
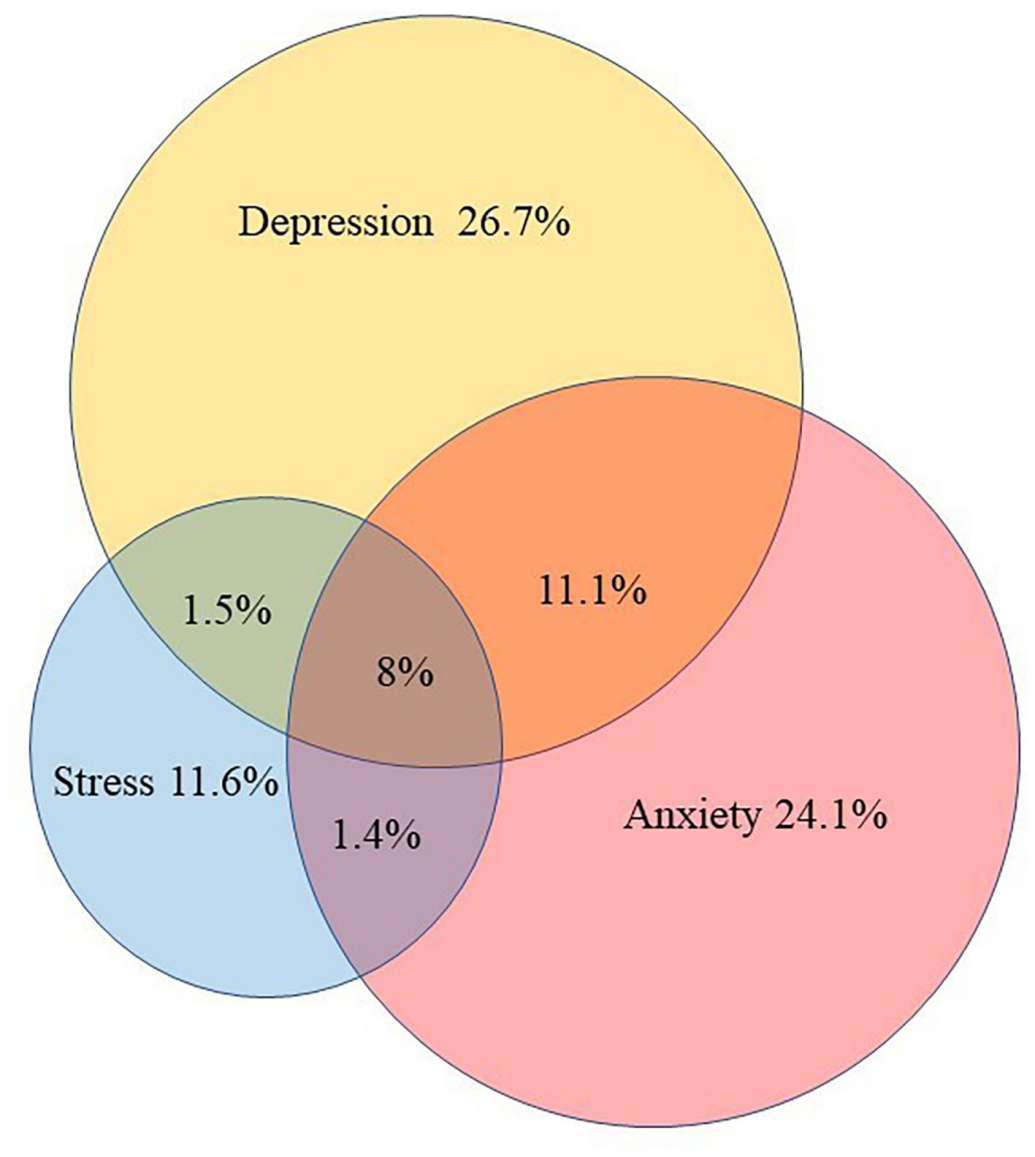
Prevalence of probable depressive, anxious and stress symptoms. Normal participant, 66.32% (2509), either symptoms of depression, anxiety or stress 33.68% (1274). Depressive symptom, 26.7% (1011); anxious symptom, 24.1% (913); stress symptom, 11.6% (416); depressive and anxious symptoms alone, 11.1% (375); depressive and stress symptoms alone, 1.5% (44); anxious and stress symptoms alone, 1.4% (39); all of three symptoms, 8% (304).

The average of DASS-depression, DASS-anxiety, and DASS-stress subscales were 13.43 [standardized deviation (SD) = 7.76], 9.53 (SD = 7.47), and 13.80 (SD = 7.56), respectively. Figure 1 presents the prevalence of probable depressive, anxious, and stress symptoms. Based on the cut-off scores for DASS-21, 33.68% of the participants experienced mental health symptoms. The rates of participants with probable depressive, anxious and stress symptoms were 26.7% (95%CI = 25.3–28.1%), 24.1% (95%CI = 22.8–25.5%), and 11.6% (95%CI = 10.1–12.0%), respectively. In addition, 11.1% (95%CI = 10.1–12.2%) experienced both depressive and anxious symptoms, 1.4% (95%CI = 1–2%) experienced both anxious and stress symptoms, and 1.5% (95%CI = 1.1–2.0%) experienced both depressive and stress symptoms. Overall, 8% (95%CI = 7.0–8.7%) experienced all three symptom domains.

### Univariable Analysis

In univariable analysis, significant differences were found in age, sex, marital status, education level, monthly income, number of night shifts per month, working years, perceived impact of the COVID-19 on medical work, and the QOL score between the depression and no-depression groups (all *P* < 0.05 except working years), between anxiety and no-anxiety groups (all *P* < 0.05), and between stress and no-stress groups (all *P* < 0.05). However, there was no significant difference in frontline experience with COVID-19 patients between the three groups (Table 1).

### Multivariable Analysis

Table 2 presents the results of binary logistic regression analysis. Those aged 30–39 years (OR = 1.5, *P* = 0.03), of male gender (OR = 1.2, *P* = 0.04), single (OR = 1.44, *P* = 0.01), and/or had negative perceptions of the impact of COVID-19 on healthcare (OR = 2.34, *P* < 0.001) were more inclined to experience depressive symptoms. Psychiatrists who were divorced and widowed (OR = 1.56, *P* = 0.03), worked more than 4 night shifts per month (OR = 1.33, *P* < 0.001), had longer working years (OR = 1.71, *P* < 0.001), and/or had endorsed a negative perceived impact of COVID-19 on healthcare (OR = 2.05, *P* < 0.001) were more inclined to experience anxious symptoms. In addition, psychiatrists with high QOL scores had lower odds of experiencing depressive, anxious, and stress symptoms (all *P* < 0.001) (see Table 2 for details).

**Table 2:** Multivariable logistic regression analysis of depressive, anxious and stress symptoms in 3,783 Psychiatrists in China.

	**Depression**			**Anxiety**			**Stress**		
	**B**	***P***	**OR (95% CI)**	**B**	***P***	**OR (95% CI)**	**B**	***P***	**OR (95% CI)**
**Age (ref. ≤29)**									
30–39	0.41	0.03^**^	1.5 (1.03–2.16)	0.16	0.40	1.18 (0.80–1.70)	0.50	**0.04** ^**^	1.65 (1.02–2.67)
40–49	0.23	0.32	1.25 (0.80–1.96)	−0.05	0.82	0.94 (0.60–1.49)	0.24	0.43	1.27 (0.70–2.30)
≥50	0.11	0.66	1.11 (0.69–1.79)	0.02	0.93	1.02 (0.63–1.65)	0.24	0.46	1.27 (0.66–2.43)
**Sex (ref. female)**	0.18	**0.04** ^**^	1.20 (1.01–1.42)	0.05	0.56	1.05 (0.88–1.25)	0.14	0.25	1.14 (0.90–1.44)
**Marital status (ref. Married)**									
**Single**	0.37	**0.01** ^**^	1.44 (1.1–1.88)	0.18	0.21	1.19 (0.90–1.57)	0.43	**0.02** ^**^	1.53 (1.07–2.17)
**Divorced and widowed**	0.28	0.19	1.32 (0.86–2.01)	0.45	**0.03** ^**^	1.56 (1.03–2.36)	0.57	**0.04** ^**^	1.77 (1.03–3.01)
**Education level (ref. College degree/medical degree only)**									
Add on master degree	−0.45	<0.001^***^	0.64 (0.52–0.78)	−0.27	**0.01**	0.76 (0.62–0.93)	−0.15	0.31	0.86 (0.65–1.14)
Add on doctorate degree	−0.37	0.097	0.69 (0.44–1.07)	−0.002	0.99	0.99 (0.64–1.54)	0.106	0.73	1.11 (0.61–2.01)
Monthly income (RMBs)	−0.32	<0.001^***^	0.73 (0.63–0.84)	−0.51	<0.001^***^	0.60 (0.51–0.70)	−0.40	<0.001^***^	0.67 (0.53–0.83)
Number of night shifts per month (ref. ≤ 4 times)	0.13	0.19	1.14 (0.94–1.38)	0.29	<0.001^***^	1.33 (1.10–1.61)	0.26	**0.046** ^**^	1.29 (1.01–1.66)
**Working years (ref. ≤5 years)**									
6–10 years	–	–	–	0.19	0.27	1.20 (0.86–1.68)	−0.17	0.43	0.85 (0.56–1.28)
≥11 years	–	–	–	0.54	<0.001^***^	1.71 (1.20–2.45)	0.06	0.81	1.05 (0.67–1.66)
**Perceived impact of the COVID-19 on medical work (ref. Minimal impact)**									
Negative	0.85	<0.001^***^	2.34 (1.86–2.95)	0.72	<0.001^***^	2.05 (1.62–2.59)	0.88	<0.001^***^	2.39 (1.81–3.18)
Positive	−0.26	<0.001^***^	0.77 (0.64–0.93)	−0.04	0.70	0.96 (0.79–1.16)	−0.02	0.86	0.97 (0.74–1.27)
**Quality of life**									
General QOL	−0.90	<0.001^***^	0.41 (0.35–0.48)	−0.57	<0.001^***^	0.56 (0.48–0.65)	−0.48	<0.001^***^	0.61 (0.52–0.74)
General health status	−0.45	<0.001^***^	0.64 (0.56–0.72)	−0.58	<0.001^***^	0.56 (0.49–0.63)	−0.81	<0.001^***^	0.45 (0.38–0.53)

*OR, Odds ratio; CI, confidence interval; Ref., reference group, COVID-19 Coronavirus Disease 2019*.

*Bold values*:

** *P < 0.05;*

*** *P < 0.001*.

### Multivariate Analysis of Variance (MANOVA)

The MANOVA revealed that depressive symptoms were significantly associated with general QOL [*F*_(1, 3775)_ =42.266, *P* < 0.001] and general health status [*F*_(1, 3775)_ =11.807, *P* < 0.001]. Anxious symptoms were significantly associated with the general QOL [*F*_(1, 3775)_ =7.13, *P* < 0.001] and general health status [*F*_(1, 3775)_ =17.647, *P* < 0.001]. Stress symptoms were also significantly associated with general QOL [*F*_(1, 3775)_ =12.58, *P* < 0.001] and general health status [*F*_(1, 3775)_ =40.261, *P* < 0.001]. Furthermore, the interaction of depressive × anxious × stress symptoms was also significantly associated with general QOL [*F*_(4, 1936)_ =5.663, *P* < 0.001] and general health status [*F*_(4, 2788)_ = 5.663, *P* < 0.001].

## Discussion

This is the largest study conducted to date on the psychological wellbeing of psychiatrists in the context of the pandemic and the first investigation performed in Asia. Findings from an online survey of a large (*N* = 3,783), nationally representative sample of psychiatrists in China revealed the following. First, the prevalence of probable depressive, anxious, and stress symptoms among Chinese psychiatrists was high, 26.7, 24.1, and 11%, respectively. More than 10% (11.1%) reported both depressive and anxious symptoms and 8% experienced all three symptoms during the COVID-19 pandemic. Second, with regard to sociodemographic and pandemic-related factors, psychiatrists who were aged 30–39 years, male, single or divorced or widowed, with a lower level of education and lower levels of income, and those who worked more night shifts per month and who had more work years were more inclined to report depressive or anxious or stress symptoms. In addition, those who endorsed more perceived negative impact of the COVID-19 pandemic on healthcare and poorer QOL were more prone to report symptoms of depression, anxiety, and stress. Finally, there was a bidirectional association between mental health symptoms and QOL among psychiatrists in China.

### Prevalence of Depressive, Anxious, and Stress Symptoms

In the systematic review and meta-analysis, Sahebi et al. (2021) reported that the prevalence of depression and anxiety in the healthcare workers during the COVID-19 pandemic was 24.83 and 24.94%, respectively; these percentages are consistent with this study (corresponding 26.7 and 24.1%). However, this study reported higher prevalence rates of depressive (26.7%) and anxious (24.1%) symptoms among Chinese psychiatrists than in the corresponding studies (18.4% depression and 13.3% anxiety) in Chinese medical staff (including 40.6% doctors and 59.4% nurses) during the pandemic (Liu et al., 2021) and in Chinese psychiatric medical staff (17.4% depression) before the COVID-19 pandemic (Hu et al., 2020). This may relate to sample differences in measurements, professions, and/or regions. Another possible explanation is that the epidemic dramatically influenced Chinese psychiatrists' mental wellbeing. Additionally, other scholars recently reported that depressive, anxious, and stress symptoms of healthcare workers ranged from 38.4 to 58%, 25.8 to 54.2%, and 37.9% to 55.1%, respectively, all of which were higher than our results (Xiao et al., 2020; Zhou et al., 2020; Campos et al., 2021). This difference may be because this study was executed after these aforementioned investigations; hence, there may have been understandable improvements in the psychological health of healthcare workers over time. Of note, the prevalence of all three symptoms (8%) among Chinese psychiatrists who specialize in interventions for individuals with mental illness in this study was similar to that of the general population in at least one other country (Ghana) (8.3%) (Amu et al., 2021), suggesting that caring for this patient population may be detrimental to the mental health and wellbeing of psychiatrists. Such a high prevalence of mental health symptoms among Chinese psychiatrists means that more easily accessible and uniquely tailored healthcare services should be provided to this population.

### Associated Factors for Depressive, Anxious, and Stress Symptoms

Most previous studies (Lai et al., 2020; Xiao et al., 2020; Peng et al., 2021; Li et al., 2021a) found that women were more vulnerable to depressive and anxious symptoms during the COVID-19 pandemic, which contrasts with our results. However, like our study, Alnazly et al. (2021) reported that being male was associated with greater psychological distress among healthcare workers during the pandemic. One possible explanation for the inconsistency is that men had higher unemployment (Matthay et al., 2021) and greater economic stress during the outbreak. Furthermore, female doctors in psychiatry may be better at using their expertise to care for themselves psychologically than their male counterparts and medical staff in other specialties. In terms of sociodemographic factors, however, consistent with several previous reports (Ridley et al., 2020; Zhou et al., 2020; Schmitt et al., 2021), we found that participants who were younger, single, had more night shifts per month, and had longer working years and/or low-level income were more inclined to experience mental health symptoms.

We did not replicate findings from several prior studies (Rossi et al., 2020; Zhang et al., 2020; Zhou et al., 2020) that frontline healthcare workers were more prone to report mental health symptoms than those not caring directly for patients diagnosed with COVID-19. This may reflect the fact that the majority of psychiatrists had more access to mental health training than medical staff in other departments and disciplines (Lima et al., 2020; Mattila et al., 2021) and thus may be better prepared for the tremendous pressures associated with providing frontline healthcare services (Dal Santo et al., 2020). Future health workers should be required to receive mental health training. Previous studies have reported negative psychological effects of the COVID-19 pandemic on medical staff (including burnout, compassion fatigue, and moral injury) (Meynaar et al., 2021; Su et al., 2021). On the contrary, Byrne et al. (2021) indicated that the COVID-19 pandemic improved healthcare working environments by increasing the staffing of doctors, offering more access to senior clinical support, and supporting accelerated clinical decision-making by physicians. However, our study reported that up to 45.5% of the psychiatrists viewed the pandemic as having neither a negative nor a positive impact on their medical work. One possible explanation is that psychiatrists are accustomed to enduring stressful work schedules and attending to vulnerable patients with psychiatric disorders. Of note, however, in this study, we did find that 12.8% of Chinese psychiatrists perceived that COVID-19 had a negative impact on their medical work, such that they were intending to leave and change careers. Future research should shed light on these vulnerable healthcare workers and increase efforts to provide them much needed mental health support.

An additional finding of note is that poor QOL was an independent risk factor for depressive, anxious, and stress symptoms. This result is consistent with previous research demonstrating that high levels of depressive and anxious symptoms correlated with reduced levels of QOL and health status in different individuals (Stark et al., 2002; Johnston et al., 2019; Phillips et al., 2020), such as concussion symptoms (Doroszkiewicz et al., 2021), Parkinson's disease (Lo Buono et al., 2021), older adults (Wu et al., 2021), and ischemic stroke (Huang et al., 2010).

### Bidirectional Relations Between Depressive, Anxious, Stress Symptoms, and QOL

In this study, we found that depressive, anxious, and stress symptoms were independently related to general QOL and general health status. In addition, our findings are in concordance with previous studies showing that high levels of perceived stress are not conducive to QOL (Altunan et al., 2021). In this study, tests of between-subjects effects showed that the depression × anxiety × stress interaction had an effect on general QOL and general health status. This result is consistent with prior reports in which depressive and anxiety disorders were co-morbid in at least half of the patients who had either diagnosis (Davey et al., 2017) and that the co-occurrence of depression and anxiety had markedly lower overall QOL (Li et al., 2021b). Therefore, we emphasize the bidirectional relation between depressive, anxious, and stress symptoms on the one hand and QOL on the other hand.

Of note, unlike most other studies (Suarez et al., 2018; Yao et al., 2021; Yen et al., 2022), this study only used two items (i.e., general quality of life and general health state) instead of the four domains (i.e., physical health, psychological, social relationships, and environment) to evaluate QOL. Our findings support the feasibility of these two separate items in assessing the overall QOL (Haag et al., 2017; Li et al., 2021b). Future studies to replicate our findings in different samples may be needed.

### Strengths and Limitations

In this study, we provide relatively comprehensive, valid, and reliable data. The tertiary hospitals involved in this study come from almost all provinces and autonomous regions in mainland China (except Gansu and Tibet). Thus, the results are likely generalizable throughout the country. In addition, the online anonymous survey attracted a large number of participants and generated good response rates, yielding representative information.

Nevertheless, several limitations need to be considered. First, depressive, anxious, and stress symptoms are not clinical diagnoses based on the Diagnostic and Statistical Manual of Mental Disorders, fifth edition, abbreviated as DSM-5, or the International Classification of Diseases, 10th revision, abbreviated as ICD-10. Rather, the questionnaire responses reflect trends of mental health problems in the past week. Second, other factors that might be associated with depressive, anxious, and stress symptoms, as well as QOL, such as burnout (Murat et al., 2021), smoking cigarettes (Fluharty et al., 2017), or insomnia (Shanahan et al., 2014), were not examined. Third, the cross-sectional study design limits our ability to track participants' mental health problems and QOL, which may have shifted in response to the changing pandemic landscape. In addition, the cross-sectional design precludes us from making causal interpretations. Fourth, this study used only two separate items to assess the overall QOL, which may be considered less comprehensive as the ones that used the four-domain instrument (Suarez et al., 2018; Yao et al., 2021; Yen et al., 2022), and it may also limit the generalizability of our findings. Finally, due to differences in work environment and exposures between diverse medical specialties and across countries, the generalizability of the findings to other populations deserves further investigation.

## Conclusion

In conclusion, during the COVID-19 pandemic, nearly one-fourth of psychiatrists in China reported symptoms of depression and anxiety, and more than 10% of psychiatrists reported stress symptoms. There was a high level of comorbidity among the three mental health symptom clusters in Chinese psychiatrists. We also found that mental health issues are associated with poor QOL. In addition, 12.8% of psychiatrists still experience the negative impact of the COVID-19 on their healthcare work. Therefore, to improve the mental health system in China, more attention and awareness need to be directed to the psychological wellbeing of the psychiatric workforce. This must include efforts to ensure early detection and increase the accessibility and effectiveness of mental health interventions for psychiatrists in the face of crises, such as the COVID-19 pandemic.

## Data Availability Statement

The original contributions presented in the study are included in the article material; further inquiries can be directed to the corresponding authors.

## Ethics Statement

The studies involving human participants were reviewed and approved by the Ethics Committee of Chaohu Hospital of Anhui Medical University. The patients/participants provided their written informed consent to participate in this study.

## Author Contributions

HL, FJ, and Y-lT were the guarantor and designed the study. ML, LX, YY, LZ, TL, and YL participated in the acquisition, analysis, and interpretation of the data. ML drafted the initial manuscript. SZ, Y-lT, and NK revised the article critically for important intellectual content. All authors contributed to manuscript revision, read, and approved the submitted version.

## Funding

This study was supported by the National Clinical Key Specialty Project Foundation (CN) and the Beijing Medical and Health Foundation (Grant No. MH180924).

## Conflict of Interest

The authors declare that the research was conducted in the absence of any commercial or financial relationships that could be construed as a potential conflict of interest.

## Publisher's Note

All claims expressed in this article are solely those of the authors and do not necessarily represent those of their affiliated organizations, or those of the publisher, the editors and the reviewers. Any product that may be evaluated in this article, or claim that may be made by its manufacturer, is not guaranteed or endorsed by the publisher.
